# Patients preference for mode of delivery in a Middle Eastern society: a cross-sectional study

**DOI:** 10.3389/fmed.2025.1688528

**Published:** 2025-11-06

**Authors:** Nashwa Aldardeir, Mohammed Sulaimani, Sarah Y. Bahowarth, Aseel H. Abdulaziz, Firyal K. Alghalayini, Khloud W. Halabi, Anas S. Alyazidi, Eman S. Jastanieh, Raghad Alharbi, Shahad T. Alamoudi, Meshari Alrumaihi, Mohammed A. Malibary

**Affiliations:** 1 Department of Obstetrics and Gynecology, Faculty of Medicine, King Abdulaziz University, Jeddah, Saudi Arabia; 2 Faculty of Medicine, King Abdulaziz University, Jeddah, Saudi Arabia

**Keywords:** cesarean section, mode of delivery, maternal health, knowledge, opinion

## Abstract

**Background:**

Cesarean section (CS) rates are increasing globally, including in the Middle East. While CS can be a lifesaving procedure, unnecessary CS can lead to complications for both mother and child. Understanding factors influencing women’s delivery preferences is crucial for optimizing maternal and child health outcomes.

**Methods:**

This cross-sectional study investigated factors influencing the preferred mode of delivery among 661 pregnant women in Jeddah, Saudi Arabia. Data were collected using a structured questionnaire and analyzed using descriptive statistics and logistic regression.

**Results:**

Multiple factors showed significant associations with CS preference in multivariable analysis. Non-Saudi women had substantially higher odds of preferring CS compared to Saudi nationals (aOR 2.45, 95% CI 1.30–4.60). Urban residency (aOR 2.89, 95% CI 1.45–5.78) and higher income ≥50,000 SAR (aOR 4.15, 95% CI 1.02–16.88) were also strong predictors. Conversely, women aged 31–40 years had significantly lower odds of preferring CS (aOR 0.23, 95% CI 0.07–0.78) compared to those aged 18–24. Women with no prior CS were more likely to prefer spontaneous vaginal delivery, and previous CS in private hospitals significantly increased CS preference for subsequent deliveries.

**Conclusion:**

This study highlights the complex interplay of demographic, socioeconomic, and personal factors influencing women’s delivery preferences. Understanding these factors is crucial for developing targeted interventions to promote informed decision-making and optimize maternal and child health outcomes. Further research is needed to investigate the underlying reasons for these preferences.

## 1 Introduction

Cesarean section (CS) is the most frequent obstetric operation designed to treat or avoid serious complications that could endanger the lives of the mother or fetus (1). Globally, CS rates have been witnessing a surge due to multiple reasons. In middle- and high-income countries, studies reported an increase in CS rates despite lack of evidence of improvement toward maternal and perinatal mortality and morbidity (2), rather, some data suggest the contrary and propose a greater risk for complications when undergoing CS including maternal mortality, vesical injury, ureteral tract injury and hysterectomy (3–5). Furthermore, unnecessary CS can be associated with increased surgical complications, abnormal placentation in subsequent pregnancies, uterine rupture, and longer recovery times (3–5). In contrast, spontaneous vaginal delivery (SVD) offers several health benefits, including faster maternal recovery, establishment of breastfeeding, transfer of beneficial microbiota to the newborn, and reduced risk of respiratory complications in infants (4, 5). However, while CS is associated with increased maternal morbidity, it may offer benefits in specific contexts including reduced risk of urinary incontinence, pelvic organ prolapses, and avoidance of emergency procedures in complicated labors. A balanced understanding of risks and benefits is essential for shared decision-making (6). Nonetheless, it is important to note that the World Health Organization (WHO) recommends an ideal cesarean section rate of 10–15%, noting that rates above this threshold are not associated with reduced maternal or neonatal mortality.

Regionally, the Middle East has mirrored this global trend with rising CS rates across many countries. Within Saudi Arabia specifically, this pattern is particularly evident, with healthcare research increasingly concentrating on the risks associated with different delivery methods. While CS can be lifesaving in certain circumstances, it carries specific fetal risks including respiratory distress syndrome, iatrogenic prematurity, altered immune development, and increased likelihood of neonatal intensive care unit admission compared to vaginal delivery (7). Studies also highlighted an increase in CS, which is like the global surge, including one study in the United Arab Emirates, which presented a rate of CS exceeding the average global rates (8). Similarly, a single-center study in the densely populated Egypt said that CS contribute more than half of deliveries annually (9). Furthermore, a substantial increase has been observed over a decade in Saudi Arabia for the CS according to public estimates, which reach up to 80% (10). This is echoed by single-center data, which similarly present an increase in the rates of CS (11). Midst these data and the conflicting reports on the outcome of CS, patients’ choices constitute one of the key reasons for unnecessary CS procedures made in the absence of clear medical indications (12). Several studies shed light on factors influencing women and contributing to this decision, which varied between parity history as well as some country-specific characteristics, and others (13–16). Meanwhile, none of them quantitatively summarized the results (13–16).

Giving birth is one of the most important and profound human experiences with high individual significance (7). Women who give birth often express feelings of empowerment, joy, and accomplishment, especially after giving birth naturally without the need for medical assistance (17). Multiple factors influence delivery method preference including maternal age, education, previous delivery experience, cultural beliefs, fear of labor pain, perceptions of safety, and healthcare provider recommendations. Understanding these determinants is crucial for developing appropriate patient education and counseling strategies. Moreover, while previous Saudi studies have documented rising CS rates, few have comprehensively examined the interplay of socioeconomic, obstetric, and psychological factors shaping women’s preferences in the Jeddah region. This study aims to identify modifiable determinants of delivery preference to inform targeted interventions and address the growing CS rate in this specific population.

## 2 Materials and methods

### 2.1 Study design and setting

We conducted a cross-sectional exploratory study in Jeddah, Saudi Arabia, following the Strengthening the Reporting of Observational Studies in Epidemiology (STROBE) guideline (18). The study utilized a multi-center approach across major public and private hospitals between January and December 2024 to investigate patients’ preferences for mode of delivery. We included both pregnant women (≥28 weeks gestation) and postpartum women (within 6 months of delivery) to capture preferences across the perinatal period. Exclusion criteria included multiple current pregnancies, known medical indications for CS (e.g., placenta previa, active genital herpes), fetal congenital anomalies, and women unable to provide informed consent. Participants were stratified by pregnancy status in analysis to account for potential differences in perspective. Data were collected using a structured, pre-validated questionnaire from a heterogeneous sample of 661 participants. The questionnaire was re-validated for our use through expert review by three obstetricians and a psychometrician, pilot testing with 30 women, and demonstrated good internal consistency (Cronbach’s alpha = 0.82). Participants were recruited during routine antenatal visits and through hospital maternity records. Enrolled women were asked their preference for delivery type during the post-delivery period. The questionnaire was distributed via secure WhatsApp links, with reminders sent at 2-week intervals to maximize response rate. To minimize response bias, we employed multiple recruitment strategies across different hospital settings, ensured anonymity of responses, and used standardized neutral language in all survey materials. The response rate was 71%, with no significant differences in basic demographics between responders and non-responders. The primary outcome, preference for mode of delivery, was assessed using the direct question with multiple response options.

### 2.2 Sampling technique

We adopted a convenience sampling method to ensure a larger cohort is enrolled in the study, with the representation of diverse demographic groups within the society under study. The sample was not restricted to certain age groups to explore potential associations and reduce the probability of confounders. The statistically appropriate sample size was calculated using Raosoft software, with a 95% confidence interval (CI) and <0.05 margin of error. The applied equation was as follows:

$n=(\text{DEFF}\times \mathrm{Np}\;(1-p))/((\begin{array}{l} d2/Z21-\alpha /2\\ \times (N-1)+p\times (1-p) \end{array}))$

where, *n* = population size, p = prevalence, d = precision (desired margin of error), DEFF = design effect, and Z1 − *α*/2 = 1.96 for a 95% confidence level. We exceeded the minimum recommended sample size, which was determined at 378 (19).

### 2.3 Data collection and variables

We obtained data through a 31-item self-administered online survey. Patients’ information was retrieved from the electronic hospital record to ensure the survey was accessible to the targeted population and subsequently contacted. For additional clarification, the first section of the survey included a consent statement explaining the nature of the study and the targeted population. The second section obtained socio-demographic data that included their gender, age, nationality, place and city of residency, marital status (e.g., single, married, divorced, or widow), certain anthropometric measurements (e.g., weight and height), educational level (e.g., uneducated, elementary, middle school, high school, university level, higher education), monthly income in local currency, and current occupation. The third section is about obstetrics and delivery, focused on current pregnancy status, number of previous pregnancies, history of live births, abortions, stillbirths, and prior delivery methods, including CS history and setting (public/private hospital). The fourth section is pain and post-delivery experience evaluated participants’ experiences with postpartum pain, duration of medication use, and perceived community awareness regarding delivery complications.

### 2.4 Data analysis

Data were cleaned, managed, and coded using Microsoft Excel 2019 (Microsoft Corporation, Redmond, WA). Statistical analysis was performed using R (RStudio, version 1.4.1106; RStudio, Inc). Descriptive analysis included frequency distributions. Cross-tabulations were evaluated using the chi-square test, and odds ratios (OR) with 95% confidence intervals (CI) were calculated. Multivariable logistic regression included covariates selected *a priori* based on literature review: age, nationality, education, income, parity, previous CS, and source of delivery information. Missing data (<5%) were handled using multiple imputation. Model assumptions were checked using residual analysis and variance inflation factors (all <2.0). A *p*-value of less than 0.05 was considered statistically significant.

## 3 Results

### 3.1 Sociodemographic characteristics

**Figure 1:**
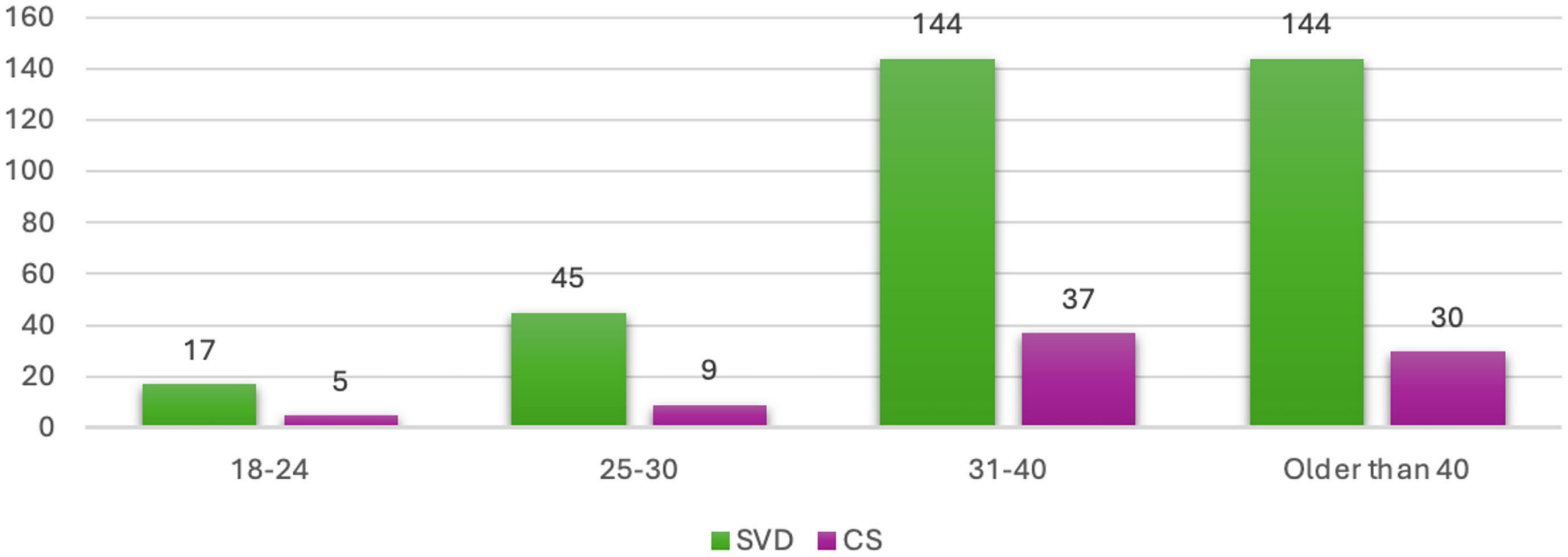
Preferred mode of delivery according to age groups.

The sociodemographic characteristics of the 661 participants are summarized in Table 1. The cohort was predominantly Saudi (79.4%), married (62.5%), and resided within Saudi Arabia (92.9%), with a majority living in the study city of Jeddah (63.2%). The age distribution was broad, with the largest proportion of participants falling within the 31–40 year age group (29.0%). Education levels were high, as most participants had attained a university degree (61.9%). Nearly half of the participants (49.6%) reported a monthly income of less than 5,000 Saudi Riyals. Regarding anthropometrics, the majority of participants were within the 150–169 cm height range (86.4%) and the 50–69 kg weight category (52.8%; Figure 1). This profile describes a cohort that is broadly representative of an urban patient population in the region.

**Table 1:** Sociodemographic characteristics of participants and association with preferred mode of delivery.

Variable	Group	Total (*N* = 661)	% of Total	SVD (*N* = 354)	CS (*N* = 82)	Total (SVD+CS)	*p* value
Age group	<18 years	25	3.8%	4	1	5	0.912
	18–24 years	179	27.1%	17	5	22	
	25–30 years	79	12.0%	45	9	54	
	31–40 years	192	29.0%	144	37	181	
	>40 years	186	28.1%	144	30	174	
Nationality	Saudi	525	79.4%	297	57	354	**<0.05**
	Non-Saudi	136	20.6%	57	25	82	
Place of residence	In Saudi	614	92.9%	334	81	415	0.091
	Out of Saudi	47	7.1%	20	1	21	
City of residence	In Jeddah	418	63.2%	251	74	325	**<0.05**
	Out of Jeddah	243	36.8%	103	8	111	
Marital status	Married	413	62.5%	-	-	-	-
	Single	225	34.0%	-	-	-	-
	Divorced	16	2.4%	-	-	-	-
	Widow	7	1.1%	-	-	-	-
Educational level	Middle school	7	1.1%	5	1	6	0.843
	Elementary	2	0.3%	2	0	2	
	Higher education (Master’s, PhD)	125	18.9%	91	18	109	
	High school	117	17.7%	39	7	46	
	University	409	61.9%	216	56	272	
	Uneducated	1	0.2%	1	0	1	
Monthly income (SAR)	<5,000	328	49.6%	140	27	167	**<0.05**
	5,001-10,000	105	15.9%	70	11	81	
	10,001-20,000	142	21.5%	95	22	117	
	20,001-50,000	71	10.7%	47	18	65	
	>50,000	15	2.3%	2	4	6	
Height category	<150 cm	34	5.1%	-	-	-	-
	150–159 cm	284	43.0%	-	-	-	-
	160–169 cm	287	43.4%	-	-	-	-
	170–179 cm	31	4.7%	-	-	-	-
	180–189 cm	21	3.2%	-	-	-	-
	≥190 cm	4	0.6%	-	-	-	-
Weight category	<50 kg	97	14.7%	-	-	-	-
	50–69 kg	349	52.8%	-	-	-	-
	70–89 kg	173	26.2%	-	-	-	-
	90–99 kg	18	2.7%	-	-	-	-
	≥100 kg	24	3.6%	-	-	-	-

Bold values represent *p*-values. Statistical significance was set at *p* < 0.05.

### 3.2 Factors influencing preferred mode of delivery

The association between participant characteristics and their preferred mode of delivery is presented in Table 1. In the bivariate analysis, several demographic factors showed significant associations with a preference for CS. These included non-Saudi nationality (*p* < 0.05), residence within Jeddah city (*p* < 0.05), and higher monthly income (*p* < 0.05). In contrast, age group (*p* = 0.912) and educational level (*p* = 0.843) were not significantly associated with delivery preference.

Further analysis of obstetric history, detailed in Table 2, revealed that the number of previous live births was a significant factor (*p* < 0.05). However, other obstetric factors such as current pregnancy status, history of stillbirth, total number of pregnancies, previous abortion, and the age of the youngest child did not demonstrate a significant influence on the choice between SVD and CS.

**Table 2:** Factors affecting preferred mode of delivery.

Variable	Group	SVD (*N* = 354)	CS (*N* = 82)	Total	*p* value
Are you pregnant now?	No	325	77	402	**0.524**
	Yes	29	5	34	
Live births category	None	29	3	32	**< 0.05**
	One	57	11	68	
	Two	64	29	93	
	Three	72	19	91	
	Four or More	132	20	152	
Stillbirths category	None	237	54	291	**0.175**
	One	54	16	70	
	Two	32	10	42	
	Three or More	31	2	33	
Total number of pregnancies	0	13	1	14	**0.390**
	1	45	10	55	
	2	52	19	71	
	3	62	18	80	
	4	72	14	86	
	5	38	9	47	
	6	26	6	32	
	7	18	3	21	
	>7	28	2	30	
Previous abortion	No	205	48	253	**0.917**
	Yes	149	34	183	
Age of youngest child	None	34	3	37	**0.348**
	One month to 2 years	244	60	304	
	6–10 years	1	0	1	
	>10 years	75	19	94	

Bold values represent *p*-values. Statistical significance was set at *p* < 0.05.

### 3.3 Impact of CS on delivery mode preferences

The relationship between CS history and participants’ preferred mode of delivery reveals several significant findings (Table 3). The number of previous CS strongly influenced delivery preferences, with participants who had no prior CS more likely to prefer SVD (*p* < 0.05). Additionally, those who had CSs in private hospitals were more inclined to choose CS for subsequent deliveries (*p* < 0.05). Elective CS on request also played a significant role, with participants who opted for elective CS more likely to continue preferring CS (*p* < 0.05). In contrast, factors such as whether the CS was due to medical indications and the number of previous SVD did not significantly affect delivery preferences (*p* > 0.05).

**Table 3:** Cesarean section history and preferred mode of delivery.

Variable	Group	SVD (*N* = 354)	CS (*N* = 82)	Total	*p* value
Have you ever had a cesarean section?	No	210	40	250	**0.082**
	Yes	144	42	186	
Number of cesarean sections	0	210	38	248	**< 0.05**
	1	68	17	85	
	2	30	15	45	
	3	25	6	31	
	4	15	4	19	
	5	6	1	7	
	6	0	1	1	
Where did you do a cesarean section?	Did not have a cesarean section	204	36	240	**< 0.05**
	Public hospital	60	14	74	
	Private hospital	90	32	122	
Is your cesarean section due to medical indications?	Did not have a cesarean section	204	36	240	**0.073**
	No	16	4	20	
	Yes	134	42	176	
Electively on request cesarean section?	Did not have a cesarean section	204	35	239	***< 0.05***
	No	129	36	165	
	Yes	21	11	32	
How many vaginal deliveries have you had?	0	102	28	130	**0.383**
	1	46	13	59	
	2	46	16	62	
	3	62	13	75	
	4	47	5	52	
	5	18	2	20	
	6	22	3	25	
	7	5	2	7	
	8	2	0	2	
	>8	4	0	4	

Bold values represent *p*-values. Statistical significance was set at *p* < 0.05.

### 3.4 Opinions on mode of delivery and associated factors

Table 4 presents opinions on the mode of delivery and associated factors among participants who preferred SVD and CS. The decision-making process regarding the mode of delivery was similar between the groups, with mothers and doctors playing key roles (*p* = 0.203). A significant majority believed that SVD has fewer complications for the mother compared to CS (*p* < 0.05). However, opinions on complications for the baby did not significantly differ between SVD and CS (*p* = 0.285).

**Table 4:** Opinions on mode of delivery and associated factors.

Variable	Group	SVD (*N* = 354)	CS (*N* = 82)	Total	*p* value
Who has the right to choose the mode of delivery?	Mother	148	28	176	**0.203**
	Doctor	206	54	260	
Which method has fewer complications (for the mother)?	SVD	327	70	397	**< 0.05**
	CS	27	12	39	
Which method has fewer complications (for the baby)?	SVD	217	45	262	**0.285**
	CS	137	37	174	
If your delivery was SVD, how long did the pain last after birth (in days)?	One day	43	9	52	**0.588**
	Two days	22	9	31	
	Three days	35	7	42	
	Four days	10	4	14	
	5 days	18	5	23	
	Six days	7	2	9	
	Seven days	55	9	64	
	More than seven days	61	9	70	
	Did not have a natural birth	103	28	131	
If your delivery was CS, how long did the pain last after birth (in days)?	One day	4	3	7	**0.387**
	Two days	2	2	4	
	Three days	11	1	12	
	Four days	5	2	7	
	Five days	10	2	12	
	Six days	2	0	2	
	Seven days	34	8	42	
	More than seven days	76	27	103	
How do you assess the awareness of complications of delivery in your community?	1 (Lowest)	70	28	98	**< 0.05**
	2	56	16	72	
	3	131	27	158	
	4	52	3	55	
	5 (Highest)	45	8	53	
What is your source of information about pain relief during childbirth?	Internet	92	29	121	**0.160**
	Hospital midwife	13	5	18	
	Antenatal classes	11	3	14	
	Opinion of friends	14	1	15	
	Opinion of family	43	3	46	
	Physician anesthesiologist	11	2	13	
	Obstetrician-gynecologist	161	35	196	
	Professional literature	9	4	13	
In your opinion, what is the most useful non-pharmacological method for pain relief during labor?	Breathing techniques	189	37	226	**0.317**
	Hypnosis	10	0	10	
	Immersion in water	31	11	42	
	Massage	58	14	72	
	Physical activity	45	15	60	
	TENS	3	0	3	
	Upright position	18	5	23	
How long did you use medication to control pain after SVD (in weeks)?	Less than a week	78	22	100	**0.921**
	One week	55	15	70	
	Two weeks	26	6	32	
	Three weeks	9	3	12	
	Four weeks	6	0	6	
	More than one month	3	1	4	
	Did not use any medications	96	24	120	
How long did you use medication to control pain after CS (in weeks)?	Less than a week	25	4	29	**< 0.05**
	One week	37	11	48	
	2 weeks	20	15	35	
	Three weeks	13	4	17	
	Four weeks	10	4	14	
	More than one month	12	3	15	
	Did not use any medications	156	30	186	

Bold values represent *p*-values. Statistical significance was set at *p* < 0.05.

When assessing post-delivery pain duration, there were no significant differences in how long the pain lasted after SVD or CS (*p* = 0.59 and *p* = 0.39, respectively). However, community awareness of delivery complications was perceived as low, with a significant difference noted across groups (*p* < 0.05). The internet and obstetrician-gynecologists were the most common sources of information about pain relief during childbirth, although this was not statistically significant (*p* = 0.16). Breathing techniques were the most favored non-pharmacological method for pain relief during labor, with no significant difference observed between the groups (*p* = 0.32).

Regarding pain management after delivery, the duration of medication use was similar following SVD (*p* = 0.92), while after CS, there was a significant variation, with some participants requiring medication for more than a month (*p* < 0.05). These findings reflect the different perspectives and experiences related to SVD and CS, particularly in terms of perceived complications and pain management.

### 3.5 Significant factors influencing preferred mode of delivery

A multivariable logistic regression was performed to identify independent predictors of a preference for Cesarean Section (CS), with the full results detailed in Table 5. The analysis revealed a distinct profile of women with a higher likelihood of preferring CS. Demographically, women aged 31–40 years had significantly lower odds of preferring CS compared to the 18-24-year reference group. Conversely, non-Saudi nationality was a strong predictor, associated with more than double the odds of preferring CS compared to Saudi nationals. Socioeconomic and residential factors were also significant. Residing outside of Jeddah was associated with a substantially lower likelihood of preferring CS. Furthermore, a monthly income exceeding 50,000 SAR was the strongest socioeconomic predictor, significantly increasing the odds of a CS preference. Finally, obstetric history played a role; women with two children had significantly higher odds of preferring CS compared to those with four or more children.

**Table 5:** Logistic regression model coefficients.

Predictor	OR (95% CI)	*p* value
Age group		
31–40 vs. 18–24	0.23 (0.06–0.93)	**0.039**
Older than 40 vs. 18–24	0.24 (0.06–1.02)	**0.053**
Nationality		
Non-Saudi vs. Saudi	2.29 (1.19–4.38)	**0.013**
City of residence		
Out of Jeddah vs. In Jeddah	0.31 (0.12–0.76)	**0.010**
Monthly income (Saudi Riyal)		
>50,000 vs. 10,001-20,000	12.64 (1.11–143.67)	**0.041**
Live births category		
Two Children vs. Four or More	2.73 (1.28–5.79)	**0.009**

Bold values represent *p*-values. Statistical significance was set at *p* < 0.05.

## 4 Discussion

The study aimed to identify the preferred mode of delivery and factors influencing women preferring CS and SVD in a tertiary care center in Jeddah, Saudi Arabia. The study involved surveying women who were attending antenatal care appointments at our center. After collecting and analyzing the data, several factors and novel findings were presented in this study. Women’s autonomy can lead to a positive birth experience, and it is considered an essential part of quality maternity care (20, 21). During this significant life stage, many women desire autonomy to make their own choices about their healthcare and the well-being of their unborn child, to feel empowered to take an active role in their pregnancy journey, and to make decisions that align with their personal values and beliefs and the expected health outcome for their fetus (22, 23). Therefore, it is important to investigate factors that influence their decision.

### 4.1 Influence of demographics on delivery preferences

Specific demographic factors were analyzed to evaluate their impact on delivery preferences. Age was found not to be a significant factor in determining the preferred mode of delivery in our study. This result aligns with a previous study conducted in Saudi Arabia, which also found no correlation between age and delivery preference (24). However, a study in Poland reported that older women were more likely to prefer cesarean delivery, with a mean age difference of 1.25 years between those opting for CS and SVD (25).

Nationality emerged as a significant factor, with Saudi women showing a higher preference for SVD, while non-Saudi women preferred CS. This finding corroborates previous research, which also indicated a higher prevalence of CS preference among non-Saudi women (26). Additionally, residents in Jeddah and women with higher incomes were more likely to prefer CS. Notably, a prior study at King Abdulaziz Hospital in Jeddah found a preference for SVD among participants, suggesting a shift in attitudes over the past 2 years (27). As for the income variable, the wide confidence interval suggests caution in interpretation due to limited sample size in this subgroup.

### 4.2 Impact of CS history on delivery preferences

**Figure 2:**
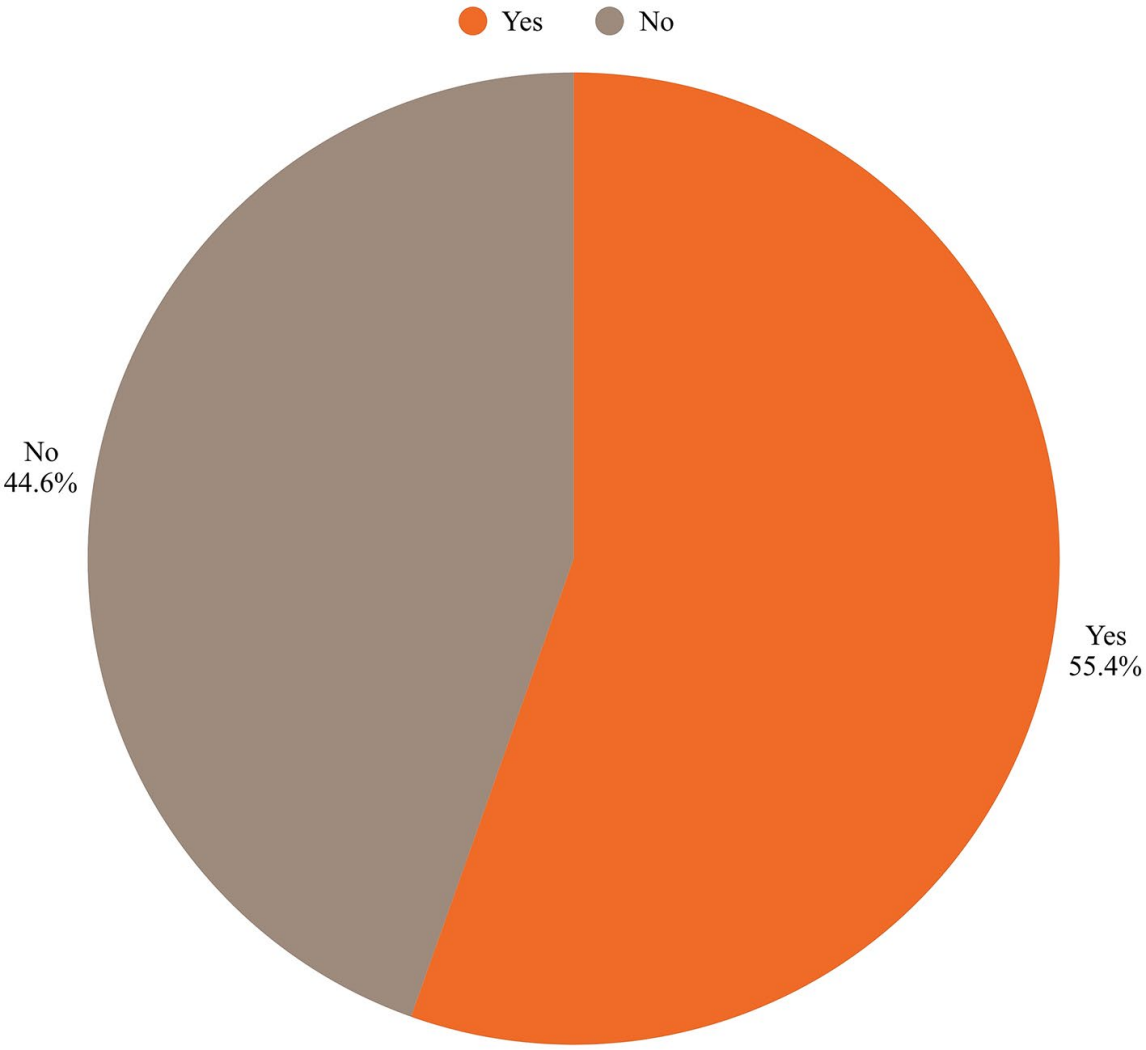
Percentage favoring spontaneous vaginal delivery.

A significant finding of our study was the influence of previous CS on delivery preferences. Women with no prior CS were more likely to prefer SVD (*p* < 0.05) as seen in Figure 2. This trend is consistent with a study by Ana-man-Torgbor et al. (28), which found that most women with prior CS preferred SVD, with only 10.4% opting for CS (29). Similarly, research by Gbaranor et al. concluded that SVD was the preferred mode of delivery, with only 2.9% of participants expressing a preference for CS (30). However, a study by Zewude et al. reported a higher prevalence of CS preference (25%), which exceeds the national average (31).

Our study also highlighted the role of private hospital deliveries in shaping future preferences. Women who underwent previous CS in private hospitals were significantly more likely to prefer CS for subsequent deliveries (*p* < 0.05). This finding aligns with research by Rayhan, which emphasized the role of private healthcare facilities in influencing CS preference due to perceived superior quality of care (32). Similarly, Mazzoni et al. found that women delivering in private hospitals were more likely to opt for CS, even among those who initially preferred SVD (17).

### 4.3 Logistic regression analysis of factors influencing preferences

Logistic regression analysis identified several factors associated with delivery preference. Women aged 31–40 years had significantly lower odds of preferring CS compared to those aged 18–24 years. This finding contrasts with some literature that reports increased CS preference among older women due to concerns about pregnancy complications (33). The observed pattern in our cohort may reflect unique cultural or generational attitudes toward childbirth in our setting, though the cross-sectional design precludes determination of causal mechanisms.

Nationality also influenced delivery preferences, with non-Saudi women showing a higher likelihood of opting for CS. While previous studies have explored factors contributing to rising CS rates in Saudi Arabia, including educational status and income (34), further research is needed to investigate the specific differences between Saudi and non-Saudi women. Similarly, a study from Bangladesh identified maternal education, previous CS, and socioeconomic status as key determinants of cesarean delivery (35). Our findings regarding CS preferences take on added significance in light of recent evidence on CS-related complications. A recent regional study by Abdulrazzak et al. confirmed high CS prevalence and identified similar demographic risk factors in neighboring populations, reinforcing the regional pattern our study observed (36). Furthermore, the long-term maternal complications associated with CS, including isthmocele and subsequent infertility as highlighted by Al-Ghotani et al., underscore the importance of minimizing non-medically indicated procedures (37). From the neonatal perspective, while rare, serious complications such as portal vein thrombosis following CS delivery, as documented by Haddad et al., contribute to the comprehensive risk–benefit assessment that should inform delivery method decisions (38).

Urban residency and income levels were additional factors influencing preferences. Women residing in urban areas, particularly Jeddah, and those with higher income were associated with greater odds of preferring CS, consistent with patterns observed in other middle-income settings (39). Additionally, this finding aligns with research indicating that urban women have greater access to healthcare facilities and are more exposed to information about delivery options, which may influence their decision-making (31). Higher-income women may also perceive CS as a safer or more convenient option, especially when private healthcare services are readily accessible.

### 4.4 Sources of information and pain management preferences

Our study also revealed critical insights into women’s sources of information and perceptions. Notably, most participants perceived community awareness of delivery complications as low. The internet and obstetrician-gynecologists were the most common sources of information about pain relief, while breathing techniques were the most favored non-pharmacological method. These findings highlight a significant opportunity to enhance patient education through trusted digital platforms and to integrate training on non-pharmacological pain management, like breathing techniques, into routine antenatal counseling.

### 4.5 Clinical and policy implications

The findings of this study suggest several actionable strategies for clinical practice and public health policy including targeted education by developing evidence-based educational programs addressing misconceptions about CS, particularly for higher-income women, urban residents, and non-Saudi women. These programs should be delivered through popular channels like digital platforms and reinforced by healthcare providers. Structured antenatal counseling can be valuable by integrating structured discussions of delivery preferences, including a balanced view of risks and benefits for both CS and SVD, into routine antenatal care visits. Also, training healthcare providers in shared decision-making and effective communication is needed to ensure women’s choices are informed and aligned with clinical evidence and the incorporation of training on non-pharmacological pain management methods, such as breathing techniques, into antenatal classes to empower women and reduce fear of SVD.

### 4.6 Limitations

This study has several limitations that should be considered when interpreting the results. Its cross-sectional design precludes the establishment of causal relationships between the identified factors and delivery preferences. The reliance on self-reported data introduces the potential for recall bias, particularly among postpartum women reflecting on past decisions, and social desirability bias. Although the study utilized a multi-center approach, the use of convenience sampling and its restriction to a major urban center (Jeddah) may limit the generalizability of the findings to rural populations or other regions of Saudi Arabia with different healthcare landscapes and cultural norms. Furthermore, the online, self-administered nature of the survey might have excluded women with lower literacy levels or limited digital access, potentially leading to an underrepresentation of disadvantaged socioeconomic groups.

Despite these limitations, the study possesses notable strengths. The large sample size provides robust statistical power for the analyses conducted. The inclusion of a wide range of socioeconomic, demographic, and obstetric factors allows for a comprehensive exploration of determinants influencing delivery preference. Furthermore, the focus on a specific, understudied urban population in Jeddah yields valuable insights that are directly relevant to local health policy and clinical practice, providing a foundation for targeted interventions to align delivery method choices with evidence-based care,

## 5 Conclusion

In conclusion, our study identified nationality, income level, urban residence, and previous delivery experience as key determinants of delivery method preference among women in Jeddah. Non-Saudi women, those with higher incomes, and urban residents showed significantly greater preference for cesarean delivery, while women without prior cesarean sections predominantly preferred spontaneous vaginal delivery. These findings highlight the need for targeted educational interventions and shared decision-making approaches in antenatal care to ensure delivery method choices align with clinical evidence and individual patient values.

## Data Availability

The raw data supporting the conclusions of this article are available from the corresponding author upon reasonable request, subject to patient confidentiality considerations.
