# Correction: Patients preference for mode of delivery in a Middle Eastern society: a cross-sectional study

**DOI:** 10.3389/fmed.2025.1745213

**Published:** 2025-11-27

**Authors:** Nashwa Aldardeir, Mohammed Sulaimani, Sarah Y. Bahowarth, Aseel H. Abdulaziz, Firyal K. Alghalayini, Khloud W. Halabi, Anas S. Alyazidi, Eman S. Jastanieh, Raghad Alharbi, Shahad T. Alamoudi, Meshari Alrumaihi, Mohammed A. Malibary

**Affiliations:** 1 Department of Obstetrics and Gynecology, Faculty of Medicine, King Abdulaziz University, Jeddah, Saudi Arabia; 2 Faculty of Medicine, King Abdulaziz University, Jeddah, Saudi Arabia

**Keywords:** cesarean section, mode of delivery, maternal health, knowledge, opinion

The funder KAU Endowment (WAQF) at King Abdulaziz University, Jeddah, Saudi Arabia was erroneously omitted. The correct funding statement reads

The author(s) declare that financial support was received for the research and/or publication of this article. The project was funded by KAU Endowment (WAQF) at King Abdulaziz University, Jeddah, Saudi Arabia.

In the acknowledgements the sentence “The authors acknowledge with thanks WAQF and the Deanship of Scientific Research (DSR) for technical and financial support.” was erroneously omitted.

The original version of this article has been updated.

